# Imaging blood-brain barrier dysfunction: A state-of-the-art review from a clinical perspective

**DOI:** 10.3389/fnagi.2023.1132077

**Published:** 2023-04-17

**Authors:** Paulien Moyaert, Beatriz E. Padrela, Catherine A. Morgan, Jan Petr, Jan Versijpt, Frederik Barkhof, Michael T. Jurkiewicz, Xingfeng Shao, Olujide Oyeniran, Tabitha Manson, Danny J. J. Wang, Matthias Günther, Eric Achten, Henk J. M. M. Mutsaerts, Udunna C. Anazodo

**Affiliations:** ^1^Department of Medical Imaging, Ghent University Hospital, Ghent, Belgium; ^2^Lawson Health Research Institute, London, ON, Canada; ^3^Department of Neurology, Vrije Universiteit Brussel (VUB), Universitair Ziekenhuis Brussel (UZ Brussel), Brussels, Belgium; ^4^Department of Radiology and Nuclear Medicine, Amsterdam University Medical Center, Amsterdam Neuroscience, Amsterdam, Netherlands; ^5^Amsterdam Neuroscience, Brain Imaging, Amsterdam, Netherlands; ^6^School of Psychology and Centre for Brain Research, The University of Auckland, Auckland, New Zealand; ^7^Centre for Advanced MRI, Auckland UniServices Limited, Auckland, New Zealand; ^8^Helmholtz-Zentrum Dresden-Rossendorf, Institute of Radiopharmaceutical Cancer Research, Dresden, Germany; ^9^Queen Square Institute of Neurology and Centre for Medical Image Computing, University College London, London, United Kingdom; ^10^Department of Molecular Imaging, Western University, London, ON, Canada; ^11^Laboratory of FMRI Technology (LOFT), USC Stevens Neuroimaging and Informatics Institute, Keck School of Medicine, University of Southern California, Los Angeles, CA, United States; ^12^Department of Medical Biophysics, Western University, London, ON, Canada; ^13^Auckland Bioengineering Institute, The University of Auckland, Auckland, New Zealand; ^14^Fraunhofer Institute for Digital Medicine, University of Bremen, Bremen, Germany; ^15^Montreal Neurological Institute, McGill University, Montreal, QC, Canada

**Keywords:** blood-brain barrier dysfunction, diagnostic imaging, magnetic resonance imaging, neurodegeneration, positron emission tomography

## Abstract

The blood-brain barrier (BBB) consists of specialized cells that tightly regulate the in- and outflow of molecules from the blood to brain parenchyma, protecting the brain’s microenvironment. If one of the BBB components starts to fail, its dysfunction can lead to a cascade of neuroinflammatory events leading to neuronal dysfunction and degeneration. Preliminary imaging findings suggest that BBB dysfunction could serve as an early diagnostic and prognostic biomarker for a number of neurological diseases. This review aims to provide clinicians with an overview of the emerging field of BBB imaging in humans by answering three key questions: (1. Disease) In which diseases could BBB imaging be useful? (2. Device) What are currently available imaging methods for evaluating BBB integrity? And (3. Distribution) what is the potential of BBB imaging in different environments, particularly in resource limited settings? We conclude that further advances are needed, such as the validation, standardization and implementation of readily available, low-cost and non-contrast BBB imaging techniques, for BBB imaging to be a useful clinical biomarker in both resource-limited and well-resourced settings.

## 1. Introduction

**FIGURE 1:**
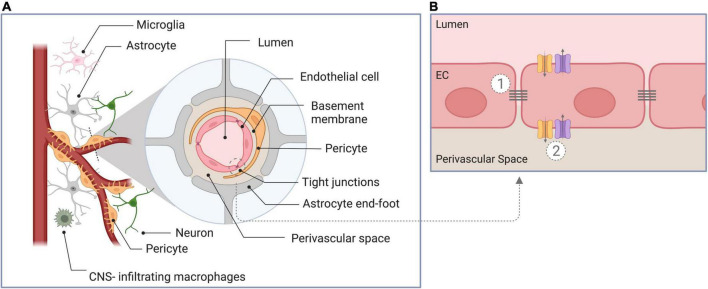
Schematic overview of components of the blood-brain barrier. **(A)** Cross-section through the neurovascular unit, consisting of endothelial cells, pericytes, astrocytes, the basement membrane, neurons, and immune cells. **(B)** Illustration of the unique properties of the blood-brain barrier endothelial cells: (1) The presence of intercellular tight junctions and the absence of fenestration limiting paracellular diffusion, and (2) the presence of specific transporters to regulate in- and efflux of substrates (transcellular transport). EC, endothelial cell.

The blood-brain barrier (BBB) is a membrane structure of the human central nervous system. The BBB is vital for maintaining brain homeostasis by regulating the exchange of compounds between the blood and the brain parenchyma (Obermeier et al., 2013; McConnell et al., 2017; Abdullahi et al., 2018). The BBB is comprised of several functional elements, represented schematically in Figure 1. Its core anatomical element is the endothelial cells (EC) lining the cerebral blood vessels. BBB ECs are unique compared to peripheral ECs as they have tightly sealed cell-to-cell contacts known as tight junctions (TJs). BBB ECs have high transendothelial electrical resistance, which significantly limits both paracellular and transcellular passage (Sweeney et al., 2018). Only molecules of less than 800 dalton (Da) are small enough to pass through TJs paracellularly (Cockerill et al., 2018). The passage of molecules larger than 800 Da is restricted to a series of specialized transporters, which dynamically regulate the transcellular in- and efflux of substrates (Obermeier et al., 2013). The endothelial monolayer is surrounded by a discontinuous layer of pericytes separated by the basement membrane. Adjacent to the pericytes are the astrocyte feet (McConnell et al., 2017; Lombardo et al., 2020), which serve as an interface between ECs and neurons. Together with immune cells (e.g., microglia and central nervous system macrophages), the aforementioned elements form the neurovascular unit (NVU).

Damage to the BBB can increase the permeability of the walls of the blood vessels within the brain, leading to the influx of neurotoxic and pro-inflammatory molecules, and invoking local inflammatory responses (Nian et al., 2020). This, in turn, further disrupts the integrity of the BBB and may lead to hemorrhagic transformation in stroke (Lakhan et al., 2013), metastatic initiation in tumors (Spronk et al., 2021), or neurodegeneration in AD (Sweeney et al., 2018). Therefore, there is growing interest in whether BBB impairment could serve as an early diagnostic and prognostic biomarker (Cockerill et al., 2018; Arba et al., 2020; Lombardo et al., 2020). However, the exact mechanisms and timing underlying BBB disruption and its role in the onset and progression of disease are not yet fully understood (Nian et al., 2020). Neuroimaging could aid in a better understanding of the factors influencing BBB dysfunction and may lead to new ways of thinking about pathogenesis and possibly treatment and prevention of neurological disorders (Lakhan et al., 2013; Cockerill et al., 2018).

This review provides an introduction to BBB imaging methods in humans by answering three key questions: (2. Disease) In which diseases could BBB imaging be useful? (3. Device) What are the currently available imaging methods for evaluating BBB integrity? (4. Distribution) And what is the potential of BBB imaging across all clinical settings including resource-limited settings?

## 2. Disease

**FIGURE 2:**
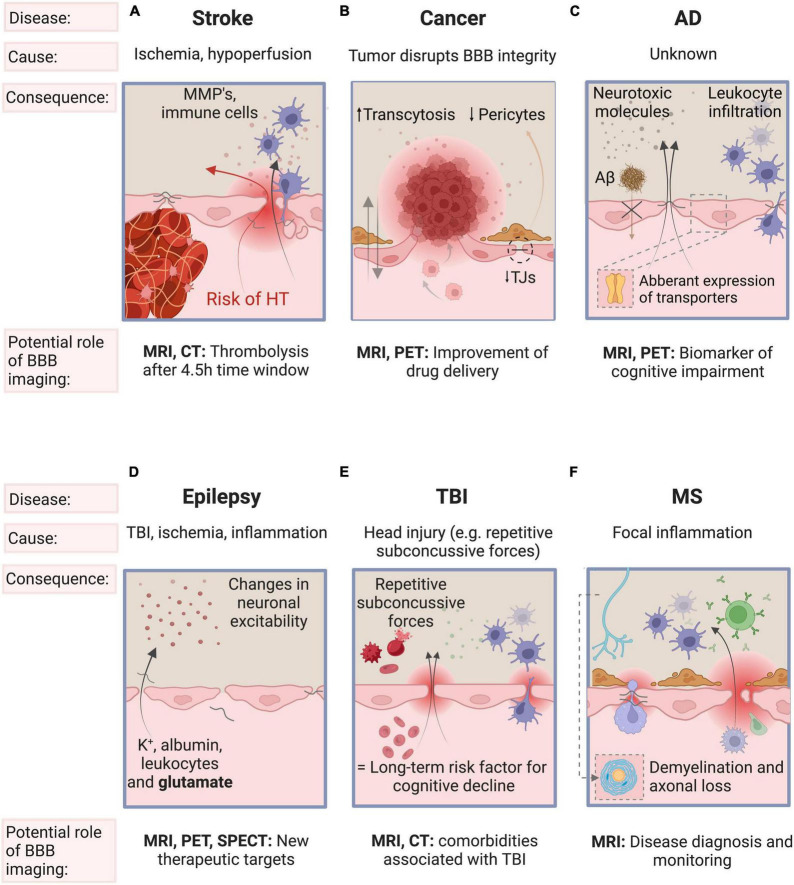
Schematic representation of the main processes driving BBB disruption, the key factors involved and the role of BBB imaging in stroke, cancer, AD, epilepsy, TBI, and MS. **(A)** BBB breakdown is initiated by ischemia and deteriorates with sustained hypoperfusion and inflammation. BBB breakdown is a precursor of more serious clinical consequences of ischemic stroke such as hemorrhagic transformation. **(B)** Brain tumors may disrupt the integrity of the BBB by the secretion of VEGF and is characterized by neurovascular decoupling, altered pericyte populations, reduction in the expression of tight junctions and changes in transcytosis mechanisms. **(C)** The initial insult leading to BBB breakdown is unknown. The resulting influx of pro-inflammatory molecules and the disturbed clearance of tau and Aβ triggers vascular-mediated secondary neuronal injury, degeneration and cognitive impairment in AD. **(D)** Epilepsy may induce BBB dysfunction, and conversely, BBB disruption may also cause (further) epileptic episodes. **(E)** Repetitive subconcussive forces and subsequent BBB dysfunction is known to be a risk factor for epilepsy and for late-life dementia (especially AD). **(F)** In MS, focal inflammation secondary to BBB disruption may trigger an inflammatory cascade leading to demyelination and axonal loss. Some features of the BBB have been omitted for simplicity (see Figure 1 for a detailed overview of the components of the BBB). AD, Alzheimer’s Disease; TBI, traumatic brain injury; MS, multiple sclerosis; BBB, blood-brain barrier; VEGF, vascular endothelial growth factor; TJ, tight junctions; Aβ, β-amyloid; K^+^, potassium.

BBB dysfunction is a common phenomenon in a number of neurological diseases where clinical evaluation with imaging can change disease management, including stroke, glioblastoma, AD, epilepsy, traumatic brain injury (TBI), and multiple sclerosis (MS). See Figure 2 for a schematic representation of the main processes driving BBB disruption in these diseases. Alterations in the BBB have also been reported in amyotrophic lateral sclerosis, Huntington’s disease, Parkinson’s disease, and depression, but are beyond the scope of this review.

### 2.1. Stroke

Ischemic stroke is characterized by an arterial occlusion that prevents viable tissue perfusion which, if not treated, can advance to brain tissue damage (Arba et al., 2020). BBB breakdown is initiated by ischemia, worsens with sustained hypoperfusion and is one of the hallmarks of ischemic stroke. Matrix metalloproteinases disrupt the BBB by degrading the TJs of ECs, leading to a significant increase in paracellular permeability. The infiltrating fluid and immune cells (neutrophils, monocytes, and T-lymphocytes) increase the BBB permeability even more. This facilitates the extravasation of peripheral blood across a disrupted BBB into the brain - also called hemorrhagic transformation (HT). HT is one of the most feared complications as it worsens outcome and increases mortality. (Lakhan et al., 2013; Abdullahi et al., 2018; Nian et al., 2020). Reperfusion is essential for brain tissue survival post ischemia. However, reperfusion therapy - also called thrombolysis - can often paradoxically contribute to additional tissue damage (reperfusion injury) and is one of the risk factors of HT (6-8% risk) (Khatri et al., 2012; Spronk et al., 2021). Reperfusion injury is characterized by oxidative stress, leukocyte infiltration, platelet activation, and BBB permeability, which may lead to brain edema, HT and eventually to neurological dysfunction [see Khatri et al. (2012)]. An important aspect for thrombolytic treatment is timing. The later recanalization is achieved, the higher the risk of HT. Therefore, current guidelines limit thrombolysis to those patients who present within 4.5 hours, to minimize the risk (Sifat et al., 2017; Bernardo-Castro et al., 2020; Nian et al., 2020). Figure 2A summarizes the main processes driving BBB disruption, the key factors involved and the role of BBB imaging in stroke, including reperfusion injury.

### 2.2. Cancer

In both primary and metastatic brain tumors, the integrity of the BBB may be altered, resulting in a more permeable vasculature known as the brain-tumor barrier (BTB). A major driver of the change in BBB architecture is tumor-secreted vascular endothelial growth factor (VEGF). It not only induces breakdown of the existing BBB, but also promotes the growth of structurally altered capillaries (abnormal expression of transporters and receptors) to meet the high metabolic demand of the tumor (Belykh et al., 2020; Mo et al., 2021).

Increased BBB permeability is a hallmark of glioma, in particular in high-grade tumors, such as glioblastomas. However, there can also be an intact BBB which can limit effective treatment by hindering homogeneous uptake of administered therapeutic agents (Arvanitis et al., 2019). This heterogeneous disruption of the BBB highlights the need to fully understand the tortuous tumor vessel architecture. In the future, combining biomarkers of BBB integrity and strategies of BBB modulation (e.g., focused ultrasound, transcranial magnetic stimulation) may allow better delivery of therapeutics across the BBB/BTB (Arvanitis et al., 2019). Figure 2B summarizes the main processes driving BBB disruption, the key factors involved and the role of BBB imaging in neuro-oncology.

### 2.3. Alzheimer’s disease

In AD, pathological changes occur years before symptoms appear. Most patients are diagnosed in the middle to late stages of the illness, when irreversible brain damage has already occurred and interventions to prevent the disease are less likely to be successful (Guest et al., 2020). One promising approach for the early, preclinical detection of AD is based upon measuring BBB integrity (Nation et al., 2019). Loss of BBB integrity in AD may result in the entry of neurotoxic and pro-inflammatory molecules as well as a reduced clearance of β-amyloid (Aβ) that may invoke local inflammatory responses, and in turn initiate multiple pathways of neurodegeneration (Sweeney et al., 2018; Hussain et al., 2021). Glucose transporters (GLUTs) are membrane proteins that facilitate glucose transport across the plasma membrane. Postmortem studies showed reductions in GLUT activity in people with AD. Evidence from human and animal AD studies shows reduced uptake of glucose into the brain, supporting these findings (Mosconi, 2013; Bouter et al., 2018; Ou et al., 2019; Chen et al., 2021). Changes in glucose metabolism may occur decades before cognitive impairment becomes apparent (Mosconi, 2013). Aquaporin 4 (AQP4) is the major water transporter in the central nervous system and is implicated in neurodegenerative diseases, such as AD (Lan et al., 2016; Silva et al., 2021). The clearance of Aβ is diminished when AQP4 activity is reduced (Xu et al., 2015).

The implementation of a combination of biomarker tests, that includes imaging measures of BBB dysfunction, could aid in early diagnosis, in monitoring disease progression and in evaluating treatment response to new drugs (Vellas, 2021). Figure 2C summarizes the main processes driving BBB disruption, the key factors involved and the role of BBB imaging in AD.

### 2.4. Epilepsy

Several reports indicated that BBB dysfunction seen in traumatic brain injury, ischemia or inflammation may contribute to epileptogenesis and facilitate seizures (Marchi et al., 2012). BBB dysfunction changes neuronal excitability due to the extravasation of potassium, albumin, leukocytes and glutamate into the brain (Shimada et al., 2014; van Vliet et al., 2015; Mendes et al., 2019). Epileptic seizures themselves may also cause BBB dysfunction, which favors seizure recurrence in epileptics (van Vliet et al., 2015; Mendes et al., 2019). Transmembrane efflux pumps pump substrates outside of cells to protect the brain from harmful substances. Two such pumps are P-glycoprotein (P-gp) and multidrug resistance-associated protein 1 (MRP1) transporters, alterations of which have been found in AD, schizophrenia, and epilepsy (Lin and Yamazaki, 2003). In epilepsy, P-gp may contribute to pharmacoresistence by limited drug distribution across the BBB. Although it is accepted that BBB dysfunction plays a key role in seizures, the exact relationship between seizures and BBB disruption is not yet fully understood. In the future, BBB imaging might facilitate identifying the key mediators involved in BBB dysfunction and may provide new therapeutic targets to better control (drug-resistant forms of) epilepsy (Han et al., 2017). Figure 2D summarizes the main processes driving BBB disruption, the key factors involved and the role of BBB imaging in epilepsy.

### 2.5. Traumatic brain injury

TBI can be classified as mild, moderate and severe (Wu et al., 2020). Whereas the diagnosis of moderate and severe TBI is readily visible on MRI (hemorrhage, increased signal intensity consistent with edema) and CT (hemorrhage), a far greater challenge is associated with the diagnosis and subsequent management of mild TBI, especially concussion which, by definition, is characterized by a normal CT (Cash and Theus, 2020). As more than 80% of TBI cases are estimated to be mild, it is particularly important to understand their pathophysiological mechanisms (Wu et al., 2020). Experimental mouse models show that mild TBI can induce microvascular injury and BBB dysfunction (Wu et al., 2020). Preliminary evidence even shows that repetitive subconcussive forces (e.g. mixed martial arts fighters and rugby players) could lead to BBB disruption (O’Keeffe et al., 2020; Verduyn et al., 2021). BBB disruption is considered an early event, occurring within hours following injury, but can persist for years. Mild TBI is therefore considered a long-term risk factor for cognitive decline and neurodegenerative diseases (Cash and Theus, 2020; Wu et al., 2020). Future BBB imaging studies may aid our understanding of comorbidities associated with TBI such as AD, post-traumatic epilepsy, and chronic traumatic encephalopathy (Cash and Theus, 2020). Figure 2E summarizes the main processes driving BBB disruption, the key factors involved and the role of BBB imaging in TBI.

### 2.6. Multiple sclerosis

MS is an autoimmune disease characterized by multifocal white matter lesions (WMLs), the so-called MS plaques (Popescu et al., 2013). BBB disruption is an early phenomenon in the formation of WMLs and is a hallmark of acutely inflamed MS lesions, as been confirmed by histopathologic examination (Choi et al., 2021). The exact trigger for BBB dysfunction is incompletely understood but the hypothesis most widely agreed upon proposes that an early focal inflammation due to activated lymphocytes traversing the BBB may trigger a complex, sustained inflammatory cascade, eventually leading to demyelination and axonal loss (Balasa et al., 2021). This BBB disruption in MS is recurrent at different time intervals and is triggered by unknown factors. When present, disruption of the BBB in WMLs leads to extravasation of gadolinium (Gd) contrast agents seen on T1-weighted MRI, highlighting “active” lesions. MRI imaging is now standard for the diagnosis of MS (Filippi et al., 2016). Figure 2F summarizes the main processes driving BBB disruption, the key factors involved and the role of BBB imaging in MS.

## 3. Device

**FIGURE 3:**
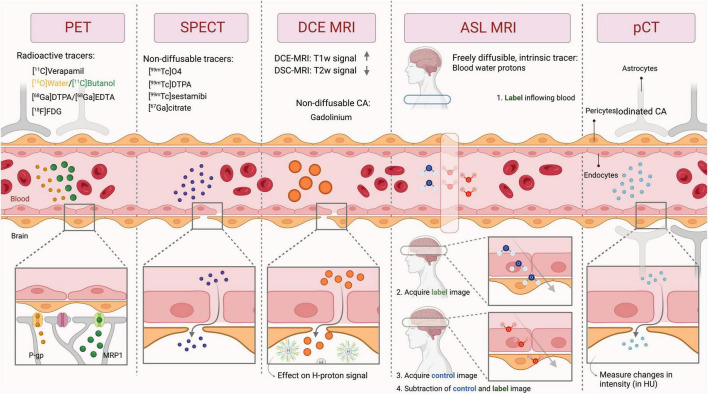
General principle of the imaging techniques used to measure BBB integrity. PET, Positron emission tomography; SPECT, Single-photon emission computerized tomography; DCE MRI, Dynamic contrast-enhanced magnetic resonance imaging; ASL MRI, arterial spin labeling MRI; pCT, perfusion computed tomography; CA, contrast agent; HU, hounsfield units; P-gp, P-glycoprotein; MRP1, multidrug resistance-associated protein 1; T1w, T1-weighted; T2w, T2-weighted.

Common BBB imaging techniques used in humans are described below, with their respective underlying principles, a description of the imaging procedure, and potential limitations. The similarities and differences between these modalities are summarized in Tables 1, 2 and Figure 3.

**TABLE 1:** Comparison of SPECT and PET imaging.

	Positron emission tomography (PET)	Single photon emission computed tomography (SPECT)
Principle	Measurement of two annihilation photons that are produced back-to-back after positron emission from a tracer, defining the line of response and an approximate tracer position.	Measurement of a single photon. Given the lack of annihilation effects, SPECT relies on the use of collimators to locate the SPECT tracer.
BBB tracers	**Increased BBB permeability**	**Non-diffusible tracers**
	[^68^Ga]DTPA, [^68^Ga]EDTA	^99m^TcO_4–_, [^99m^Tc]DTPA, [^99m^Tc]sestamibi, ^201^TI
	**Impaired glucose transport**	**Diffusible tracers**
	[^18^F]FDG	[^67^Ga]citrate, [^99m^Tc]HMPAO, Xenon-133, [^99m^Tc]ECD
	**Impaired efflux transport**	
	[^11^C]Verapamil, [^11^C]Loperamide, [^11^C]-N-desmethyl-Loperamide, [^11^C]Loperamide, [^11^C]Laniquidar, [^11^C]Tariquidar, [^11^C]Metclopramide, [^99m/94m^TC]-Sestamibi, [^18^F]Paclitaxel, [^18^F]MC225, 6-bromo-7-[^11^C]Methylpurine, 6-bromo-7-(2-^18^F-fluoroethyl)purine	
	**Dysregulated fluid exchange**	
	[^15^O]H_2_O, [^11^C]Butanol	
Safety considerations	Contrast-induced nephropathy	Contrast-induced nephropathy
Quantification BBB disruption	Absolute Quantification	Semi-Quantitative
	Kety–Schmidt model → the arterial and venous content of the tracer is plotted with time. The area between the arterial and venous curves provides the quantitative measurement of CBF.	Kety-Schmidt model, detection of signal enhancement (microsphere principle: assessment of radioactive concentration voxel by voxel - absolute or relative).
Scan duration	5-20 min	15-25 min
Attenuation correction	CT or MRI is used to provide attenuation correction information.	
Spatial resolution	2-5 mm	5-8 mm
Reproducibility	Both PET and SPECT results are very reproducible in the current standardized settings.	
Advantages	Higher sensitivity and spatial resolution, shorter scan times	Low cost, widely available, longer half-life of tracers
Challenges	Short half-life of tracers, expensive and technically demanding	Lower sensitivity and spatial resolution, longer scan times
Clinical applications	Glioblastoma, Alzheimer’s disease, epilepsy, ALS, Huntington disease, Parkinson’s disease, depression	Epilepsy, Alzheimer’s disease, multiple sclerosis, traumatic brain injury.

**TABLE 2:** Comparison of the technical specifications of DCE, DW-ASL and Multi-TE ASL.

	DCE	DW-ASL	Multi-TE ASL
Full name	Dynamic contrast-enhanced	Diffusion-weighted ASL	Multi Echo-Time ASL
Principle	**Bolus passage**	**Diffusivity**	T2/T2* relaxation
	Accumulation of contrast agent in extravascular space, causing shortening of T1 relaxation times.	Diffusion gradients separate signal from the water molecules moving more freely (blood compartment) from those in a more restrictive compartment (tissue).	Multiple echo times allows to measure the T2 time and thus separate the signal from the blood compartment (longer T2 time) and tissue compartment (shorter T2 time).
Invasiveness	Exogenous method (invasive - bolus injection of GBCA^2^)	Endogenous method (non-invasive)	Endogenous method (non-invasive)
Blood versus brain tissue	Changes in signal intensity associated with passage of contrast (GBCA decrease the T1 relaxation time when crossing the BBB)	Diffusion of vascular spins ∼ 100x diffusion tissue spins	T2 of vascular spins ∼ 2x T2 of tissue spins at 3T (T2 blood ∼ 0.275 s, T2 tissue ∼ 0.099 s)
Acquisition	T1 weighted sequence	Diffusion gradients [obtained by different b-values: e.g. 0 and 50 (Gold et al., 2021)]	Multiple echo times
Typical TR^3^/TEs^1,4^	Shortest TR < 3 s Shortest TE < 1.5 ms	TR ∼ 4.5 s TE ∼ 20-40 ms (EPI readout)	TR ∼ 4.5 s TE ∼ 14-200 ms (3D GRASE)
Total scan time	5-6 min	∼6-8 min	
Possible clinical applications	Glioma, AD^5^, epilepsy, TBI^6^, MS^7^, ALS^8^, Huntington’s disease, Parkinson’s disease, depression		

^1^Times reported refer to 3T magnetic field; ^2^Gadolinium-based contrast agent; ^3^Repetition time; ^4^Echo time; ^5^Alzheimer’s disease; ^6^Traumatic brain injury; ^7^Multiple sclerosis; ^8^Amyotrophic lateral sclerosis. T2 is the “true” T2, the transverse relaxation caused by atomic/molecular interactions, whereas T2* is the observed transverse relaxation.

### 3.1. Nuclear Imaging

For the evaluation of the BBB using radioisotopes, there are two main modalities: PET and SPECT.

#### 3.1.1. PET

##### 3.1.1.1. Underlying principles and operating procedures

**FIGURE 4:**
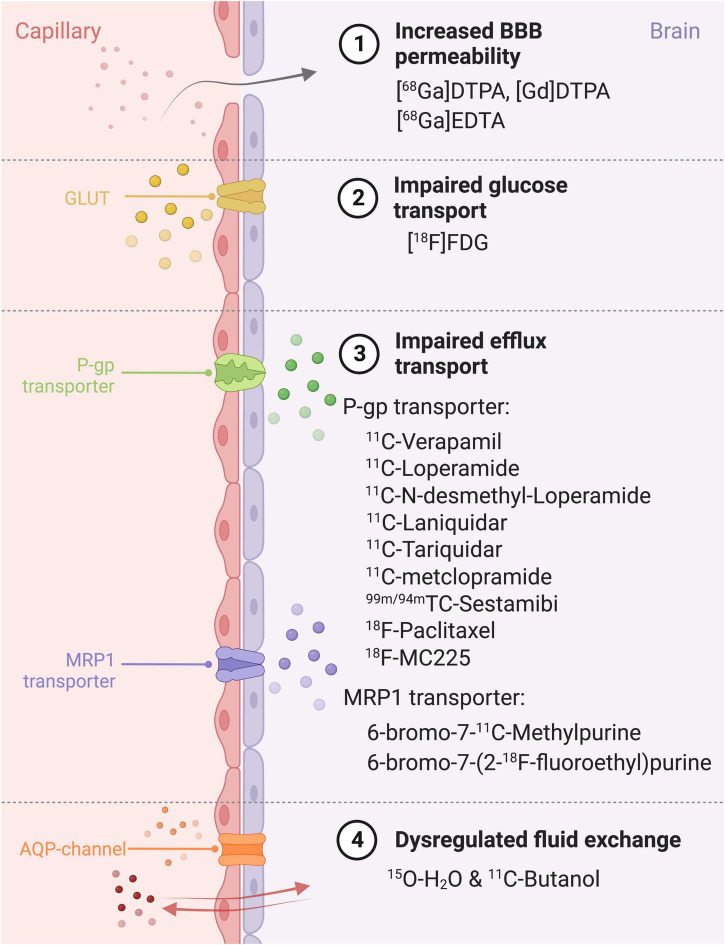
Different ways of BBB impairment, PET radiopharmaceuticals and their specific target on the blood-brain barrier. BBB, Blood-brain barrier; MRP1, Multidrug Resistance Protein 1; P-gp, P-glycoprotein; GLUT, glucose transporter; AQP, aquaporin; DTPA, diethylenetriaminepentaacetic; FDG, fluorodeoxyglucose.

PET is a molecular imaging modality, which when applied with specific radiopharmaceuticals can provide information about the degree of dysfunction of the BBB, in several neurological disorders (see section “2. Disease”). This dysfunction may include increased paracellular BBB permeability (Breuer et al., 2017), or dysfunctional transcellular transport, e.g., impaired glucose transport, impaired function of transporters and dysregulated fluid exchange. The interpretation of this data can be done qualitatively (visual analysis of tracer uptake), semi-quantitatively (maximal standardized uptake value) or quantitatively [full kinetic analysis (Box 1)]. The specific radiopharmaceuticals used to examine different aspects of the BBB are explained below and summarized in Figure 4.

**BOX 1 Glossary terms.**

**Temporal resolution**

The temporal resolution is how frequently (over a period of time) a certain imaging technique is able to capture images.

**Spatial resolution**

Spatial resolution is a measure of the smallest object that can be resolved.

**Input function**

The cumulative availability of the contrast agent in arterial plasma, measured either by serial sampling of arterial blood over a given time or extracting the mean/maximum activity from image data in vessels.

**Kinetic modelling**

Kinetic modelling is a mathematical description that described a dynamic parameter as it changes over time, typically by combining the input function of contrast agent/tracer in arterial blood and the transfer of the tracer in tissue (tissue uptake curve).

**Attenuation correction**

PET and SPECT photons that travel through the body can be absorbed or scattered (Compton scattering), the combination of these interactions is described as attenuation. Attenuation leads to underestimation of the uptake activity. Attenuation correction is a mechanism that removes tissue artifacts and is a critical step in PET/SPECT reconstruction to accurately quantify tracer distribution, especially in the brain where the bone being a dense material attenuates photons.

**T1-shortening**

T1 is the MRI time constant which describes the rate at which excited protons return to equilibrium. Gadolinium shortens the T1 time, leading to an increased signal on a T1-weighted scans. Regional signal depends on the intravascular GBCA concentration (true perfusion) on the one hand and accumulation of the GBCA in the extravascular space on the other hand (related to permeability).

**Ktrans**

Ktrans is a quantitative measure obtained using DCE MRI. It is a ‘transfer constant’ and reflects the efflux rate of Gd from blood plasma into the interstitial space.

**SNR**

Signal to noise ratio (SNR) is a measure that compares the level of a desired signal to the level of background noise. Higher SNR is synonymous with higher image quality.

**Arterial Transit Time**

Arterial transit time is defined in ASL MRI as the time that it takes for the labeled blood to flow from the labeling region to the imaged tissue.

To evaluate paracellular BBB permeability, gallium tracers can be used (e.g. [^68^Gallium]Diethylenetriamine pentaacetate, [^68^Ga]Ethylenediaminetetraacetic acid). These large molecular tracers do not cross the BBB under normal, physiological conditions. However, in epilepsy for example, insult-associated BBB leakage can be seen with gallium tracers (Breuer et al., 2017).

As described in section “2. Disease,” several transporters contribute to BBB dysfunction in a range of diseases. GLUTs may be impaired in AD, and therefore, measuring impaired glucose transport using [^18^F]Fluorodeoxyglucose ([^18^F]FDG) holds promise as a biomarker (Kyrtata et al., 2021) (see Figure 4, section “2. Disease”). P-gp function is found to be decreased in AD and increased in schizophrenia and epilepsy (Syv and Eriksson, 2013). The most frequently used P-gp substrate tracers are [^11^C]Verapamil and [^11^C]Loperamide, with others also shown in Figure 4, section “3. Device.” MRP1-transporters on the other hand have been hypothesized to play a role in AB clearance (Qosa et al., 2012; Wiese and Stefan, 2019; Storelli et al., 2021) and the PET tracers used are 6-bromo-7-^11^C-Methylpurine and 6-bromo-7-(2-^18^F-fluoroethyl)purine (see Figure 4, section “3. Device”).

**FIGURE 5:**
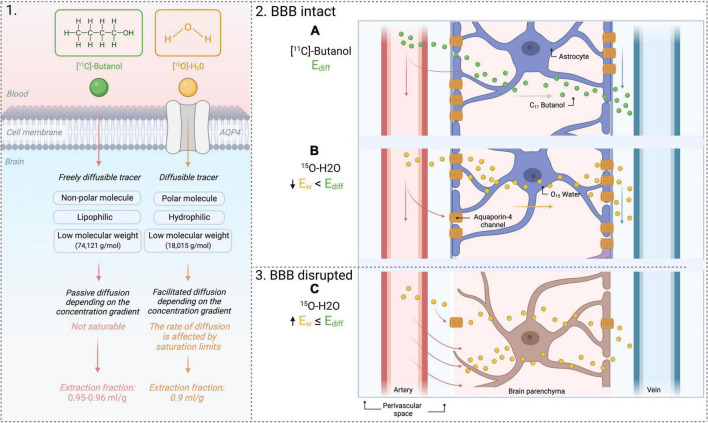
PET tracers [^11^C]Butanol and [^15^O]H_2_O. 1. Difference in extraction fraction of both tracers. 2. Use of 2 tracers to yield an index of BBB permeability. **(A)** Flow of freely diffusible tracer across an intact BBB. **(B)** Flow of [^15^O]H_2_O across an intact BBB. Water transport is modulated by AQP-4 channels. The extraction fraction of water will be lower than that of a freely diffusible tracer, [11C]Butanol. 3. Flow of [^15^O]H_2_O across a disrupted BBB. **(C)** The extraction fraction of water will increase because of water extravasation into the brain through the BBB. AQP4, aquaporin-4; E_w_, extraction fraction of water; E_diff_, extraction fraction of diffusible tracer.

Lastly, dysfunctional aquaporin channels may be imaged with PET using the combination of two tracers, [^15^O]H_2_O and [^11^C]butanol, as outlined in Figure 5. [^11^C]butanol is a freely diffusible tracer, whereas[^15^O]H_2_Os’ transport is limited to aquaporin channels (Mader and Brimberg, 2019). The first-pass extraction fraction is the fraction that enters the brain tissue during a single capillary transit. The extraction fraction is almost one (0.95-0.96 ml/g) for [^11^C]Butanol (E_*diff*_ in Figure 5). The extraction fraction for [^15^O]H_2_O (E_*w*_ in Figure 5) is slightly lower (0.9 ml/g), since water transport across the BBB is only through AQP channels (Herscovitch et al., 1987; Berridge et al., 1991; Quarles et al., 1993) (illustrated in Figure 5A). Therefore, comparing measurements of E_*diff*_ and E_*w*_ will yield an index of BBB function: in a normal BBB, E_*w*_ < E_*diff*_, but in the case of BBB dysfunction, E_*diff*_ remains the same but an increased E_*w*_ will be measured (illustrated in Figure 5B). See Herscovitch et al. (1987) for a more in-depth review of this technique.

##### 3.1.1.2. Challenges

Some PET radiopharmaceuticals that are used for BBB imaging have a relatively short half-life (i.e., ^15^O, 2 min; ^11^C, 20 min), meaning its availability will be limited to those who have an on-site cyclotron or to those who are in the vicinity of one (Duncan, 1998; Wintermark et al., 2005). For this reason, together with the fact that PET is relatively expensive and technically demanding, BBB PET imaging is used almost exclusively in large teaching hospitals and research settings. Since radiopharmaceuticals are made on demand, it is not easily available in emergency settings (e.g., acute stroke). While in most settings, the short half-life is considered a disadvantage, some perfusion protocols leverage this. The short half-life and the rapid reconstitution of the generator allows fast sequential perfusion imaging to perform repetitive studies of the BBB as described above (Carli et al., 2007). The use of serial scans with different radiotracers is limited by the risks associated with repeated exposure to ionizing radiation, but more importantly limited by the quantification approach for PET that requires serial arterial sampling of PET tracer distribution in blood for accurate quantification. However, integrated PET–MRI systems have emerged, reducing the radiation dose to the patient by eliminating CT for attenuation correction (Box 1), as well as potentially obviating the need for invasive arterial blood sampling (image-derived input function (Box 1) (Dassanayake et al., 2021; Vestergaard et al., 2021).

##### 3.1.1.3. Summary

PET is an imaging technique that enables quantification of several key biological processes in BBB dysfunction. Improved PET imaging capabilities have resulted in shorter imaging protocols, lower patient dosimetry and improved image quality. In addition, IDIF and low-dose strategies could make PET imaging less invasive and more participant friendly for adaptation in longitudinal studies. Wider adoption of PET is currently hindered because of the need of a cyclotron to perform the procedure, the inability to perform it in acute settings, the high cost of entry and operational on-going costs.

#### 3.1.2. SPECT

##### 3.1.2.1. Underlying principles and operating procedures

SPECT tracers for brain imaging are classified as either diffusible or non-diffusible (Table 1). Diffusible radiotracers — lipophilic and low molecular weight molecules such as ^99m^Tc-hexamethyl propylene amine oxime (HMPAO), Xenon-133, and ^99m^Tc-ethyl cysteinate dimer (ECD) — are capable of crossing the BBB by passive transport. They have the advantage of a quick initial brain uptake (peak within 2 min of injection) and are retained in the brain for a sufficient time (> 30 min) without redistribution, to permit image acquisition (la Fougère et al., 2009). Non-diffusible radiotracers - ions, large molecular weight or polar molecules such as^99m^ TcO4-, [^99m^Tc]DTPA, [^99m^Tc]sestamibi, ^201^TI and [^67^Ga]-citrate - are unable to cross the lipid double layer of cell membranes. Their brain accumulation, therefore, is an indication of altered BBB permeability (Bagni et al., 1983). However, BBB SPECT is still mainly reserved for research purposes and is not routinely used in the clinic.

##### 3.1.2.2. Challenges

When compared with PET, SPECT has the disadvantages of a lower sensitivity and spatial resolution (Box 1, Table 1). This is because there is no information about the direction of incoming photons when a SPECT detector registers an event. Because of the lack of annihilation effects, SPECT relies on the use of lead collimators to obtain positional information (Beyer et al., 2020). The collimator guarantees that only photons from a predefined direction are accepted. The lead collimator, however, results in a significant reduction in sensitivity. Furthermore, both sensitivity and spatial resolution are position-dependent, that is they both decrease with increasing depth in the body. Because the path length through tissue is not known, attenuation correction methods are more cumbersome in SPECT (Lammertsma, 2001). The achievable spatial resolution with the SPECT scanner is 5-8 mm full-width at half-maximum, as compared to 2-5 mm with PET (Pupi and Nobili, 2005; Catana, 2019). Despite the long scan time, there are a few reasons for the continued use of SPECT. Firstly, SPECT is more accessible, with for example five times more SPECT scanners compared with PET in North America and Europe (Israel et al., 2019). Secondly, the half life of SPECT tracers is usually longer than those of PET tracers, and thirdly SPECT has a lower cost.

##### 3.1.2.3. Summary

Although SPECT provides an assessment of the BBB in vivo and, therefore, has the potential to be a valuable research tool, it continues to suffer from poorer photon detection efficiency (sensitivity) and lower spatial resolution than PET. However, SPECT has not been completely replaced by PET because of the benefit of lower cost, greater accessibility, and the longer stability of tracers.

### 3.2. MRI modalities

The main MRI technique used to assess BBB integrity is dynamic contrast-enhanced (DCE). DCE uses gadolinium-based contrast agents (GBCA) that do not cross an intact BBB. Alternatively arterial spin labelin (ASL) measures the permeability of the BBB to water with BBB-ASL MRI (Table 2).

#### 3.2.1. Contrast-enhanced MRI techniques

##### 3.2.1.1. Underlying principles and operating procedures

DCE is one of the most widely used MRI techniques to assess BBB permeability by examining leakage of contrast agents into the brain parenchyma. GBCAs such as Gd-diethylenetriamine penta-acetic acid (Gd-DTPA), Gd-Gadoteric acid (Gd-Dota), or Gd-Gadoteridol (Gd-BT-DO3A) do not cross the intact BBB due to their large size. However, in the case of a disrupted BBB, micro-vessels become hyperpermeable and certain substances, including GBCAs, and can cross the BBB more easily. Extravasation leads to a regional signal change on the MRI image, due to the magnetic properties of GBCAs, which cause shortening of T1 relaxation times (Box 1). However, to measure BBB permeability, the movement of GBCAs from blood to the extravascular space must be tracked. To that end, multiple signal samples (e.g. images) are acquired over a 5-10 min time interval (Essig et al., 2013; Jahng et al., 2017; Stadler et al., 2017; Raja et al., 2018). The image data may be analyzed qualitatively or semi-quantitatively. Full quantification may be obtained by applying a suitable pharmacokinetic model, allowing several physiological parameters to be derived, including the transfer constant (Ktrans, Box 1), fractional plasma volume (Vp), and fractional volume of the tissue extracellular space (Ve) (Jahng et al., 2017).

##### 3.2.1.2. Challenges

DCE-MRI is used to evaluate BBB permeability, but only in pathologies where the contrast agent readily accumulates in the extracellular space, such as with brain tumors, stroke, or MS. Post-contrast signal differences are in the order of 100% or greater in tumors and around 50% in MS. Whilst in AD, with more subtle BBB dysfunction, the difference is only around 5% in the gray matter and 1-2% in white matter making it difficult to discriminate between intra- and extravascular contrast agent and to distinguish it from noise (Armitage et al., 2011; Thrippleton, 2019). To capture the slower more subtle interstitial uptake in AD, longer scan times are required to increase signal to noise ratio (SNR, Box 1) (Raja et al., 2018). Another point to consider is the spatial extent and distribution because contrary to tumors, BBB leakage is diffuse in neurodegenerative diseases (Van De Haar et al., 2017). Furthermore, despite efforts for standardization (Jain, 2013), there is still no consensus on acquisition (i.e., scanner parameters, temporal and spatial resolution and coverage) and analysis (i.e., visual inspection, pharmacokinetic or physiologic modeling) (Khalifa et al., 2014). Lastly following reports of Gd deposition in the brain (Kanda et al., 2014; Gulani et al., 2017), safety concerns have been raised, and therefore alternative, non-contrast MRI methods to study the BBB are an attractive alternative.

##### 3.2.1.3. Summary

Despite present research showing that DCE-MRI is an important clinical tool, some hurdles must be overcome. The lack of consensus on the optimal modeling approach and its low sensitivity for diagnosis of diseases characterized by subtle BBB disruption currently hinder its clinical adoption. While DCE-MRI is an established contrast-enhancement technique with routine clinical use, an emerging contrast-enhanced technique with promising clinical application worth mentioning, is chemical exchange saturation transfer (CEST). CEST is based on application of radiofrequency pluses to a selected pool of molecules at a frequency that causes loss of magnetization (saturation) of its protons to the protons of the surrounding and larger water pool (Kogan et al., 2013). This exchange of protons or ‘chemicals’ is the CEST signal that is further enhanced using exogenous agents, of which D-glucose is commonly used in indirect imaging of BBB (Elschot et al., 2021; Harris et al., 2023) - although other agents such as Salicylic Acid Analogues (Song et al., 2016) and Mannitol have been demonstrated in intra-arterial administration in rodent ischemic stroke models (Song et al., 2016; Liu et al., 2022). Challenges with obtaining the arterial input function to model glucose concentration in the parenchyma, its transport and utilization, the relatively long imaging time from application of multiple long TR saturation pulses, and importantly the lack of validation of CEST agents for BBB, present limitations its routine clinical use (Kogan et al., 2013; Harris et al., 2023).

#### 3.2.2. ASL

##### 3.2.2.1. Underlying principles and operating procedures

ASL is a non-invasive perfusion MR imaging technique, primarily used to image cerebral blood flow (CBF) (Anazodo et al., 2015), but also capable of measuring the water permeability of the BBB with a suitably adapted sequence (Günther et al., 2005; Pupi and Nobili, 2005; Grade et al., 2015, Loggia et al., 2019). ASL uses radiofrequency pulses to magnetically label blood water in the feeding brain arteries. ASL control (without labeling) and label images are then acquired after a post labeling delay (PLD), which allows the blood water to reach the brain tissue. The difference between control and label images is perfusion weighted and can be quantified as absolute CBF expressed in ml/100 g/min (Mutsaerts et al., 2020).

Modifications to the standard ASL sequence have been proposed to measure BBB permeability as blood water exchange rate across BBB by separating the intra- and extravascular fractions of the measured ASL signal (Dickie et al., 2020). The water exchange rate, kw, is the inverse of the time needed for water molecules to transfer into the tissue. BBB-ASL methods proposed to date include diffusion-weighted ASL (DW-ASL), multiple-echo-time ASL (multi-TE ASL), magnetization transfer ASL, contrast-enhanced ASL, and phase-contrast ASL (Dickie et al., 2020). From the aforementioned methods, DW-ASL and multi-TE ASL are the most used in clinical research (Lee et al., 1999; Vazquez et al., 2010; Gregori et al., 2013; Kara et al., 2013; Shao et al., 2019; Dickie et al., 2020) to date. Both rely on the physical blood and tissue properties to distinguish the intra- and extravascular water fractions. DW-ASL, utilizes the (pseudo-)diffusion coefficient of water molecules, which is approximately 100 fold larger in blood than in tissue (Shao et al., 2019). Multi-TE-based ASL exploits the difference in transverse relaxation time (T2) which is longer for blood water molecules (∼275 ms at 3T) than for water molecules in the brain tissue (∼99 ms at 3T) (Stanisz et al., 2005; Mahroo et al., 2021). See Table 2 for further technical specifications of these two ASL techniques.

**FIGURE 6:**
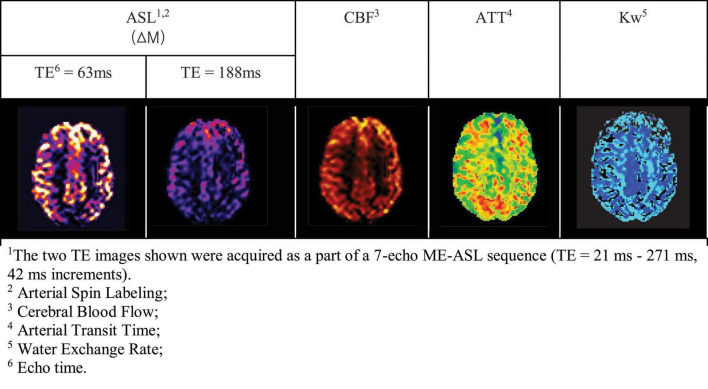
Examples of ME-ASL perfusion-weighted MRI images in a cognitively normal, 70-year-old man (Manson et al., 2020).

In both DW- and multi-TE ASL techniques, multiple parameters can be derived from the raw MRI data, including perfusion weighted images, CBF, arterial transit time and Kw. See Figure 6 as an example from a multi-TE ASL acquisition. Compared to contrast enhanced DCE-MRI, ASL may be more sensitive to subtle BBB breakdown given the smaller size of water molecules compared to gadolinium contrast agents (Shao et al., 2019; Dickie et al., 2020). However, this has not been thoroughly tested and is likely to be different depending on the disease setting (and the extent and mechanism of BBB dysfunction as shown in Figure 2).

##### 3.2.2.2. Challenges

For studying CBF, ASL has been proposed to avoid some of the disadvantages inherent to PET. ASL provides radiation-free, quantitative and non-invasive measurements and is therefore easily repeatable for longitudinally studying normal physiology or disease. However, it has an intrinsically poor SNR due to it being a subtraction technique, and labeled signal changes are low compared to contrast-enhanced MRI (Jahng et al., 2017). For multi-TE ASL, one challenge is that blood and tissue T2 values vary within subjects, due to hematocrit and oxygen saturation values. There is still no consensus on the reference values to be used (Zhao et al., 2007). Regarding DW-ASL, this technique is directionally dependent, since the diffusion gradients are applied in one direction, which might result in a less reliable estimation of BBB permeability. Moreover, DW-ASL requires data with and without vascular crushing whereas in theory multi-echo ASL does not. However, to separate vascular and extravascular tissue signal, the difference in physical properties is 100-fold in DW-Asl, and only 2-fold for multi-TE ASL (at 3T) and therefore DW-ASL may yield more robust fitting results.

##### 3.2.2.3. Summary

In general, ASL is considered a safe, completely non-invasive (using no injected contrast agent or ionizing radiation) technique and can be repeated for serial or longitudinal evaluations. The BBB mechanism underlying water exchange (Kw) and Ktrans (related to contrast agent transfer) is likely to be different (Shao et al., 2020), since the first relates to permeability to water and the latter to GBCA. The current challenge to clinical adoption is that the sensitivity of ASL to measure BBB dysfunction is still under investigation (Lin et al., 2018; Mahroo et al., 2021; Petitclerc et al., 2021).

### 3.3. X-ray modalities

#### 3.3.1. CT

##### 3.3.1.1. Underlying principles and operating procedures

Perfusion CT (pCT) is a dynamic imaging technique for measuring blood flow, blood volume, and permeability (Miles, 2004). pCT repeatedly acquires images to track an intravenous bolus of an iodinated contrast agent as it washes into and out of tissue via blood vessels and measures changes in tissue density or attenuation (in Hounsfield Units) over time (Miles, 2004; Yeung et al., 2015). The analysis of permeability can be qualitative or quantitative based on the Patlak model, which uses parenchymal contrast enhancement curves to calculate the rate of contrast transfer from an intravascular to an extravascular compartment (Dankbaar et al., 2008). In other words, permeability is measured by the rate of contrast leaving a voxel, assuming that a reduced contrast outflow (compared with inflow) is attributable to an elevated BBB permeability (Bivard et al., 2020). Major advantages of pCT are its low cost, wide availability, and simplicity (Leiva-Salinas et al., 2011). Measuring changes in contrast on CT is straightforward because of the linear relationship between signal intensity and iodine concentration. This allows the monitoring of BBB permeability over time, which is useful for evaluating the course of the disease or the effectiveness of treatment (Law et al., 2004; Leiva-Salinas et al., 2011).

##### 3.3.1.2. Challenges

Although pCT can theoretically be used to probe the BBB and seems to have several advantages, pCT is not widely used to assess BBB integrity. This is mainly because of its lack of sensitivity for subtle BBB alterations (Yeung et al., 2015). However, it has been proven useful in several other clinical applications with the most important one being stroke. Its widespread availability, speed of image acquisition, and ease of patient monitoring has made pCT part of the initial imaging assessment of stroke patients being able to identify patients who benefit from reperfusion beyond the conventional time window or in whom time of symptom onset is unknown (Petitclerc et al., 2021). These characteristics, however, are less important when evaluating BBB disruption in chronic diseases with subtle onset. A potential disadvantage of this method is the absence of standardization of the CT acquisition protocol or post processing techniques. Inaccurate post processing and interpretation can jeopardize the benefits of pCT (Dankbaar et al., 2008). Given the relative radiation burden from CT, longitudinal imaging especially in preclinical stages where the individual is otherwise considered clinically normal, justification for pCT can be challenging.

##### 3.3.1.3. Summary

pCT could theoretically be used to assess BBB integrity, but it is rarely performed. pCT lacks sensitivity to detect subtle BBB alterations, e.g. in chronic, slowly developing diseases such as dementia. However, novel spectral or dual-energy CT (Greffier et al., 2022) – imaging at high and low x-ray photon energies - particularly photon-counting CT, show promise in improving pCT sensitivity compared to conventional CT (Li et al., 2020; Greffier et al., 2022). Although the improvements in spatial resolution and noise discrimination (from lower x-ray energies) capabilities of spectral CT devices that might enable its use in BBB imaging are still an active area of technology development (Greffier et al., 2022).

## 4. Distribution

BBB imaging has a promise to be valuable in the early diagnosis of neurological disorders. This could significantly change disease management, prevention, as well as drug development. BBB imaging clinically is still limited to contrast-enhanced or nuclear medicine techniques, making it only accessible to centers where contrast agents/tracers can be readily accessed. At a time when morbidity and mortality of stroke, tumor, dementia and other neurodegenerative diseases are rising, and at a faster rate in low-to-middle income countries, access to BBB imaging - in both resource-rich and resource-limited settings - is imperative (Ezzati et al., 2018). Given the wider availability of MRI scanners compared to PET/SPECT and their higher tissue contrast compared to CT, efforts to validate and implement readily available non-contrast BBB MRI such as those based on ASL should be prioritized to bring these promising approaches to clinics worldwide. Generally, the development of ASL BBB imaging is based largely on 3T MRI scanners with research capabilities for high SNR and fast imaging including higher transmit/receive channel head coils and advanced reconstruction approaches. As such, efforts for BBB clinical translation should include implementation, validation, and standardization on 1.5T MRI scanners predominant in resource-limited settings, to expand access to BBB imaging. As the global cost of neurological diseases, particularly dementia care, continues to rise reaching a predicted US$1 trillion by 2030 (Pratchett, 2015), the need to invest in enabling clinical readiness of low-cost and contrast/radiotracer free techniques such as ASL is crucial. Even in the absence of effective treatment options, cost-effective BBB imaging approaches could provide important clinical and prognostic information and could pave the way to effective clinical drug trials by enabling selective recruitment of individuals and rapid assessment of treatment response (Robinson et al., 2013).

While the imaging techniques outlined here focus squarely on assessing BBB, some areas of the central nervous system lack BBB, such as the postrema area in the fourth ventricle of the medulla oblongata and the blood-cerebrospinal fluid barrier in the choroid plexus. Imaging these vascularized CNS structures without BBB is beyond the scope of this review.

## 5. Conclusion

In this review article, an overview of the emerging field of BBB imaging is provided for clinicians. In particular, we aimed to facilitate an understanding of technical aspects and clinical scenarios in which BBB imaging can be used. BBB imaging holds the potential to not only enable more precise and earlier diagnosis, but also to aid in selective recruitment of individuals and rapid assessment of treatment response in clinical trials. Further advances are needed, such as the validation, standardization and implementation of readily available, low-cost and non-contrast BBB imaging techniques, for BBB imaging to be a useful clinical biomarker in both resource-limited and well-resourced settings.

## Author contributions

PM: writing – original draft, visualization, and project administration. BP, JP, JV, FB, MJ, XS, OO, DW, MG, and HM: writing – review and editing. CM and TM: writing – review and editing and resources. EA: conceptualization and writing – review and editing. UA: conceptualization, writing – review and editing, resources, and supervision. All authors contributed to the article and approved the submitted version.
